# Investigation of glycerol electrooxidation activity of carbon supported PdCu bimetallic electrocatalyst

**DOI:** 10.55730/1300-0527.3506

**Published:** 2022-10-08

**Authors:** Kübranur AĞTOPRAK, Merve DOĞAN ÖZCAN, Ayşe Nilgün AKIN, Ramiz Gültekin AKAY

**Affiliations:** Department of Chemical Engineering, Faculty of Engineering, Kocaeli University, Kocaeli, Turkey

**Keywords:** Anode catalyst, glycerol electrooxidation, microwave-assisted modified polyol method, PdCu electrocatalyst

## Abstract

In this study, Pd/C, Cu/C, and a series of PdCu/C bimetallic electrocatalysts were prepared by microwave-assisted modified polyol method to determine glycerol electrooxidation reaction (GOR) activities. The effect of microwave duration on catalyst structure and GOR activity was investigated on PdCu/C bimetallic catalysts. Also, a commercial Pd/C (com-Pd/C) was used to compare catalytic activities with prepared samples. Electrocatalysts were characterized by using X-ray Diffraction (XRD), Inductively Coupled Plasma Optical Emission Spectroscopy (ICP-OES), and Transmission Electron Microscopy (TEM) analyses. In addition, the activity and stability of electrocatalysts for GOR were examined by using cyclic voltammetry (CV) and chronoamperometry (CA). In XRD results, the formation of PdCu alloy structure was observed successfully in all bimetallic catalysts. It was found that the PdCu electrocatalyst with microwave duration of 150 s (PdCu150) was exhibited homogeneous dispersion in TEM images. The particle size of 6.45 nm for PdCu150 was identified from TEM. Furthermore, the performance results were indicated that PdCu150 has the highest activity (36.02 mA/cm^2^) and stability compared to Pd/C (8.56 mA/cm^2^), and com-Pd/C (10.23 mA/cm^2^) in glycerol electrooxidation.

## 1. Introduction

In recent years, clean energy sources have attracted worldwide attention with the depletion of fossil fuel sources. Fuel cells are reported as environmentally friendly power sources for a sustainable future with high energy efficiency and clean exhaust. Hydrogen fuel cells seem promising due to their easy commercialization and high energy density. However, the limitations such as the production, storage, and transportation of hydrogen as a fuel remain to be solved [1–3]. In this regard, direct alcohol fuel cells with the electrooxidation of glycerol, ethanol, ethylene glycol, and methanol are considered as an alternative with their advantages, e.g., high energy conversion efficiencies, low emissions, easy operation, storage, and transportation [4]. Among different alcohol fuel cells, glycerol as a fuel attracts more attention due to its high reactivity, low toxicity, and high energy density. Also, it is worth mentioning that glycerol is an inexpensive by-product mostly produced on a large scale during biodiesel production. However, the catalytic properties of anode catalysts need to be improved for high glycerol electrooxidation performance and the commercialization of direct glycerol fuel cells [5–8]. Considering the above-mentioned implications, the development of nontoxic and low-cost catalysts with high catalytic activity and stability has been subjected to many studies. In literature, Pt-based catalysts are reported with high catalytic activity in glycerol electrooxidation. However, noble metals such as Pd, Au, and Ag are also studied with their reasonable activity in glycerol electrooxidation reaction (GOR) as an alternative to Pt which has scarce storage [9,10]. Furthermore, it has been reported in the literature that Pd has a remarkable electrocatalytic activity in alkaline media with low catalyst surface poisoning by adsorbed CO intermediates [1,5,11,12]. Therefore, Pd is suggested as a suitable candidate that can be an alternative to Pt. On the other hand, alloying of Pd with other metals e.g., Co, Ni, Cu, etc., could increase catalytic activity by beneficial synergistic effects and decrease the required amount of Pd [11]. Bimetallic Pd-based catalysts containing inexpensive Cu have attracted attention due to easy availability and high activity in GOR [9]. In the study of Munoz et al., Pd87Cu13/C electrocatalyst was found to be more efficient than monometallic Pd/C in alkaline media in glycerol electrooxidation. Also, glycerol electrochemical oxidation rate was found 3 times faster on Pd87Cu13/C than Pd/C indicating the presence of Cu was led to a kinetic advantage and increased the current at all potentials [13]. Wang et al. investigated the glycerol oxidation activities of Pd black and PdCu electrocatalysts. They reported that PdCu electrocatalyst was shown better results than Pd black due to its synergistic effect. This result was attributed to the increased reactivity of Cu than Pd for CO oxidation due to the stronger affinity of CO to Cu when compared to Pd. Furthermore, the d-band center of Pd was shifted down to the Fermi level indicating increased adsorbate-binding energy by the addition of Cu [14]. In another study, Rezaei et al. was investigated the activity of Cu-filled nanoporous stainless steel (NPSS) electrode with Pd and Pt in GOR. They concluded that Pt-Pd/Cu/NPSS was exhibited lower onset potential and higher current density than Pd/Cu/NPSS. The maximum current density of Pt-Pd/Cu/NPSS was determined 4.2 times higher than PdCu/NPSS electrode [5]. In the study of Maya-Cornejo et al., the PdCu/C (XC-72) electrocatalyst was synthesized by a chemical reduction method using ethylene glycol and sodium borohydride as reaction media and reducing agent, respectively. They reported that superior performance of PdCu with 3 times higher current densities in glycerol electrooxidation reaction compared to Pd/C [9]. As far as electrocatalyst preparation techniques were concerned, coprecipitation, coimpregnation, reduction in alcoholic medium, vacuum vapor deposition, surface organometallic chemistry and absorbing alloy colloids techniques were generally implemented by several researchers to prepare alloy catalysts with high surface area. In general, in an alloy or an oxide-promoted system, the first metal and the second metal or metal oxide must be in a proper interaction with each other. Hence, the activities of multicomponent catalysts are strongly dependent on the preparation method and conditions [15,16]. The polyol method was suggested to prepare nano-sized metal and alloy particles. In this method, the average diameter of the particles in the broad size range can be controlled and the mixing can be performed at molecular level. In the preparation of metal nanoparticles, the polyol method is used due to moderate reducing properties and preventing aggregation of particles during synthesis [16].

Compared with the traditional polyol method under reflux, the polyol reaction under microwave radiation has advantages such as small particle size, short reaction time, and narrow particle size distribution [17,18]. To our knowledge, Pd/C and PdCu/C catalysts prepared using microwave-assisted modified polyol method for direct glycerol fuel cell systems have not been reported in the open literature. Therefore, in the present study, monometallic Pd/C, Cu/C, and a series of bimetallic PdCu/C with different microwave duration were prepared by microwave-assisted modified polyol method. The electrocatalysts were characterized by using XRD and TEM techniques. Also, the GOR activity of electrocatalysts was determined by CV and CA analysis in an alkaline media and compared with each other and also the commercial Pd/C (com-Pd/C).

## 2. Materials and methods

### 2.1. Materials

The chemicals, Sodium hydroxide (NaOH) (Merck, for analysis, 99 %), Vulcan XC-72R carbon (Cabot Corp.), Nafion solution (Alfa Aesar, 5 wt.%), Palladium (II) acetate (PdII(OAc)_2_) (Merck, 47% Pd), Copper (II) chloride (CuCl_2_) (Merck), Glycerol (C_3_H_8_O_3_) (Tekkim, 99.5%), ethylene glycol (C_2_H_6_O_2_) (Merck, 99%), sodium borohydride (NaBH_4_) (Merck, 98%), commercial Pd/C (fuel cell store, 20 wt.% Pd) were used as received.

### 2.2. Electrocatalyst preparation

Pd/C, Cu/C, and PdCu/C electrocatalysts with different microwave duration (120 s, 150 s, and 180 s) were prepared by microwave-assisted modified polyol method. The microwave-assisted polyol method was chosen based on the studies given in the literature [13,16]. Also, the electrocatalysts were prepared in different batches at least two times for reproducibility. For all electrocatalysts, the total percentage of metal loading and Pd:Cu ratio were fixed to 20 wt.% and 1:1, respectively. Firstly, appropriate amount of palladium acetate and copper chloride were dissolved separately in 50 mL of ethylene glycol. Then 0.5 g of the Vulcan XC-72R carbon was added in 50 mL of ethylene glycol. The solutions were mixed separately for 30 min and mixed in a carbon flask. The pH was regulated to 9 with 1M NaOH solution and later NaBH_4_ solution as reducing agent was added dropwise into the solution. A microwave oven (Arçelik, MD674) was used to heat the solution with three different duration times (120 s, 150 s, and 180 s) at 700 W. After the microwave operation was finished, the final temperature of the solutions was found to be 140 °C for 120 s, 155 °C for 150 s, and 163 °C for 180 s. Aging was completed in an ice-water bath for 24 h prior to filtering and washing three times with deionized water. All samples were dried at 80 °C for 18 h. The same procedures were applied for the preparation of Pd/C and Cu/C catalysts as well. The bimetallic PdCu/C electrocatalysts were denoted as PdCu120, PdCu150, and PdCu180 according to the microwave duration of 120 s, 150 s, and 180 s, respectively. Also, the commercial Pd/C catalyst was denoted as com-Pd/C hereafter.

### 2.3. Physicochemical characterization

The electrocatalysts were characterized by the X-ray Diffraction analysis using a Rigaku Miniflexs II diffractometer equipped with a CuKα (**λ** = 0.154 nm) radiation source. The XRD spectra of electrocatalysts were collected between 2**θ** = 10**°**–80**°** at a scan rate of 2**°**/min. The morphology of electrocatalysts was determined on an Ultra High-Resolution TEM (JEOL JEM-2100) instrument at 200 kV. Inductively Coupled Plasma Optical Emission Spectroscopy (Perkin Elmer Optima 4300DV Model) was used to determine the composition.

### 2.4. Electrochemical measurements

Electrochemical experiments were conducted by GAMRY Workstation system with RDE710 rotating electrode. The coated rotating disc electrode (glassy carbon, a diameter of 5 mm) with the electrocatalyst ink was used as the working electrode. The reference electrode (Ag/AgCl) and the counter electrode (Pt) were also employed during the analysis. The preparation of the working electrode is as follows: the electrocatalyst (10 mg) was added to a nafion-water solution. Then, the ink was kept in an ultrasonic bath for 1 h to obtain homogeneity. The ink (10 μL) was dropped onto the working electrode and dried at 80 °C for 45 min, and 0.5 mg of electrocatalyst was loaded onto the working electrode. The CV curves of the electrocatalysts were recorded from −0.6 V to 0.6 V in the electrolyte solution of 0.5 M Glycerol + 0.5 M KOH with a scan rate of 50 mV/s at 850 rpm. The CA measurements were performed at −0.4 V in 0.5 M Glycerol + 0.5 M KOH electrolyte solution for 1000 s with rotating rate of 850 rpm. The analyzes were performed under continuous pure N_2_ gas flow.

## 3. Results and discussion

### 3.1. Physicochemical characterization of electrocatalysts

**Figure 1:**
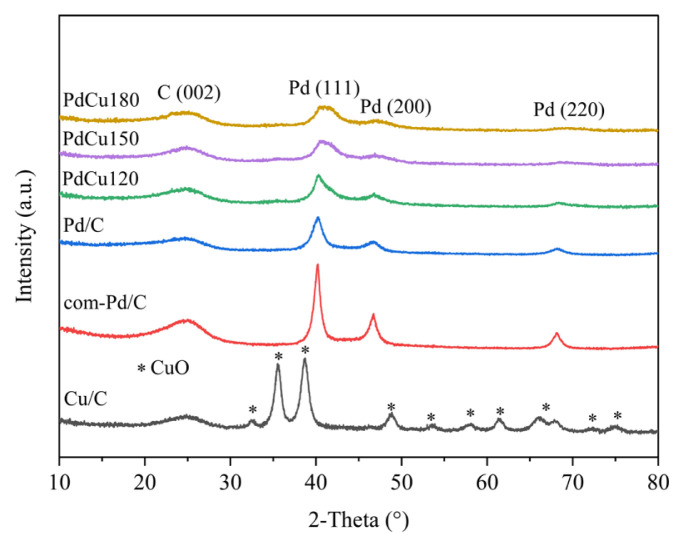
XRD patterns of electrocatalysts.

XRD patterns of electrocatalysts are shown in Figure 1. The XRD peak around 2**θ** = 24.8**°** was assigned to the face-centered cubic structure of carbon representing the C (002) plane in all samples. For monometallic Pd/C catalyst, the XRD peaks at 2**θ** = 40.19°, 46.7°, and 63.4° were referred to (111), (200), and (220) planes of face-centered cubic Pd. Moreover, 2**θ** values of the Pd (111) peaks of the bimetallic PdCu120, PdCu150, and PdCu180 were identified to be 40.40°, 40.92°, 40.97° respectively. These results were found to be in between 2**θ** values of standard Pd (111) 40.1° and standard Cu (111) 43.24° (JCPDS 03-1005) [19]. The observed shift of XRD patterns towards the positive direction indicated that Cu was added into the Pd lattice to form PdCu alloy [20]. In addition, PdO and CuO oxides were not exhibited in the catalyst structure of bimetallic samples supporting the alloy formation. For Cu/C catalyst, the formation of copper oxide phases was observed at 2**θ** = 32.45°, 35.59°, 48.77°, 53.49°, 58.01°, 61.37°, 66.11°, 72.20°, and 74.89° (COD Number 9016326) where no metallic Cu was seen.

The mean size of the crystallites was calculated utilizing the Debye Scherrer equation shown in equation (1).


(1) D=0.9λ/β cos θ

Here, D: average crystallite size (nm), **λ**: X-ray wavelength (0.154056 nm), : the maximum half peak width (FWHM) in radians, **θ**: angle of the peak [21]. In addition, the lattice parameters of crystal structure on as-prepared electrocatalysts were calculated by the Bragg equation shown in equation (2).


(2) $$\text{a}=\frac{\sqrt{2}\lambda {\text{K}}_{\text{a}1}}{\text{Sin}\theta }$$

where a is the interatomic layer distance, λK_a1_= 0.154056 is the wavelength of radiation, and θ is the angle of diffraction peak with maximum intensity [21].

The calculated structural properties of electrocatalysts are presented in Table 1. As shown, the smallest crystallite size (2.85 nm) was found on PdCu150 compared to others. The lattice parameters of bimetallic PdCu catalysts were found to be lower than Pd/C. This conclusion also indicated the formation of PdCu alloy structure in bimetallic catalysts [22].

ICP-OES analysis results of Pd/C, PdCu120, PdCu150 and PdCu180 electrocatalysts are given in Table 2. Total metal loadings (wt. %) of these electrocatalysts were reported as 19.7, 21.2, 21.3 and 20.3, respectively. The calculated atomic ratios of Pd and Cu metals in PdCu120, PdCu150, and PdCu180 electrocatalysts were found as 1.3, 1.1 and 1.2, respectively. This suggests that the ICP-OES results are in good agreement with the theoretical metallic percentages.

**Figure 2:**
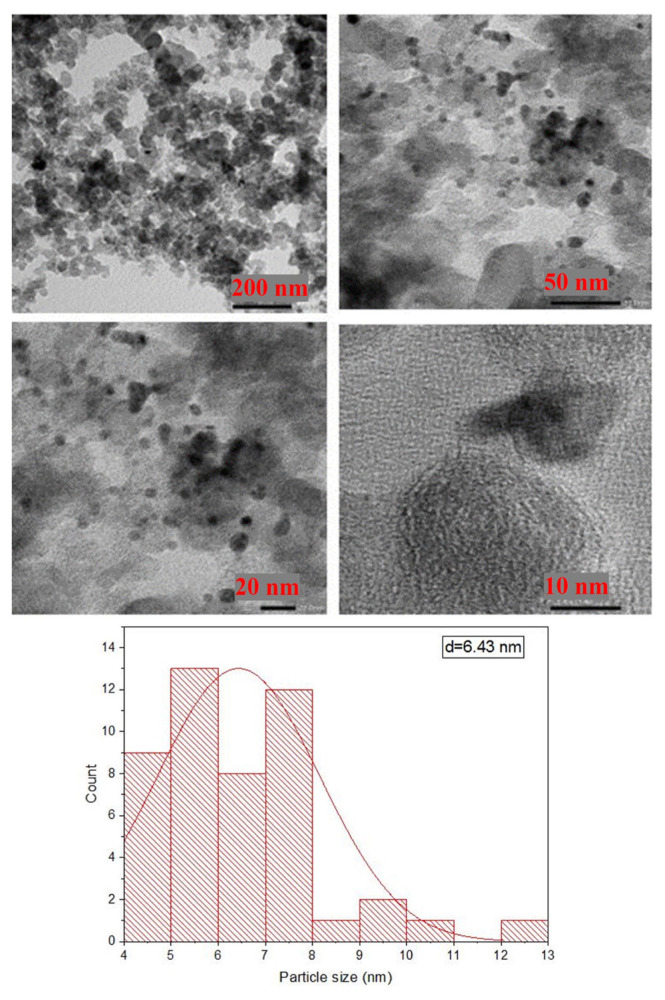
HR-TEM images and particle size distribution of PdCu150.

**Figure 3:**
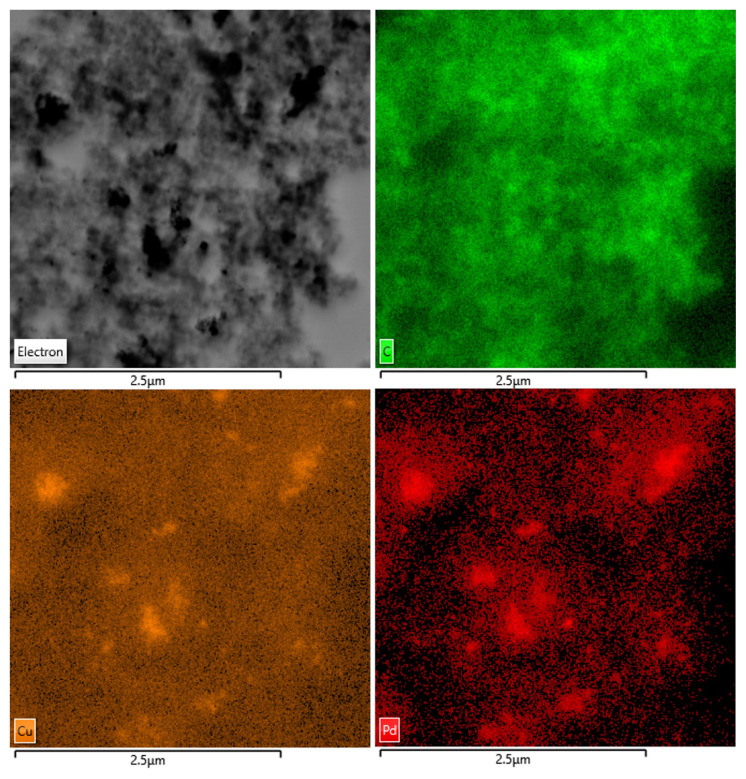
TEM mapping images of PdCu150.

The morphology and particle size distribution of the electrocatalyst (PdCu150) was determined by using TEM. The selection of PdCu150 was due to its highest GOR activity (see the section ‘Electrochemical analysis’). HR-TEM images of PdCu150 were depicted in Figure 2. In Figure 2, it was seen that spherical alloy nanoparticles were homogeneously dispersed on the carbon support. The mean particle size of the electrocatalyst was also given at 6.43 nm calculated by TEM. Additionally, TEM mapping results of PdCu150 are presented in Figure 3. As seen in Figure 3, Pd and Cu metals demonstrated a homogeneous distribution in the same regions. Hence, homogeneously dispersed PdCu nanoparticles were successfully prepared with the microwave-assisted modified polyol method.

### 3.2. Electrochemical analysis

**Figure 4:**
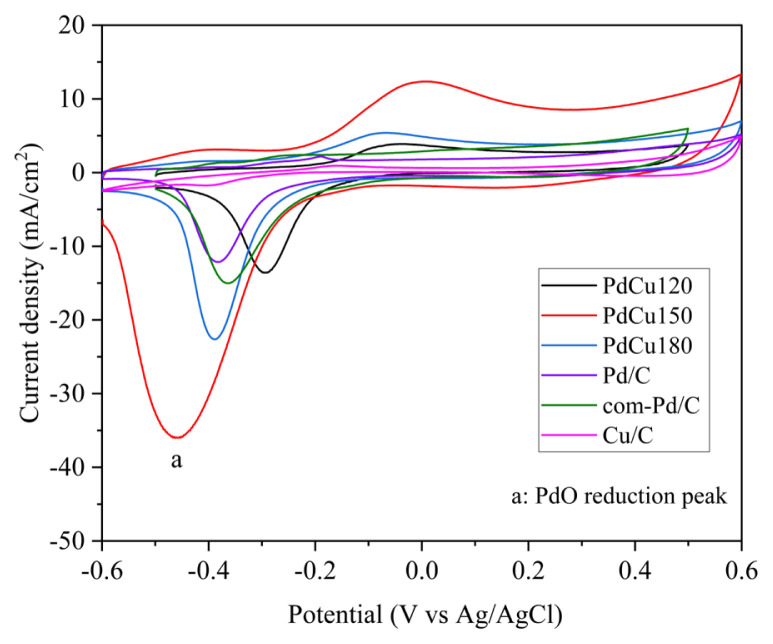
Cyclic voltammograms of electrocatalysts with a scan rate of 50 mV/s in 0.5 M KOH electrolyte solution at 850 rpm.

The electrochemical active surface area (ECSA) of electrocatalysts was determined with cyclic voltammetry (CV) by integrating PdO reduction peak [23]. Cyclic voltammograms of the electrocatalysts were recorded between −0.6 V and 0.6 V at a scan rate of 50 mV/s in 0.5 M KOH solution and given in Figure 4. As seen in Figure 4, all cyclic voltammograms demonstrated a typical oxidation peak of Pd (anodic peak) and reduction peak of PdO (cathodic peak) between −0.2 V to 0.2 V and −0.1 V to −0.6 V, respectively [5]. Meanwhile, the ECSA of the catalysts was calculated by using equation (3) and the results are summarized in Table 3.


(3) ECSA=Q/(0.405×Pd)

Where, Q is the coulombic charge (mC) estimated by the integration of peak a, Pd is the amount (mg) of palladium loaded on the electrode, 0.405 (mC/cm^2^) represents the charge needed for PdO monolayer reduction [23]. In Figure 4, when the CV curves of electrocatalysts were examined, the highest peak area of PdO reduction was obtained on PdCu150. Corollary, the highest ECSA value of 140.89 cm^2^/mg was calculated for PdCu150 compared to others as given in Table 3. This can be explained by the fact that bimetallic nanoparticles on PdCu150 were highly dispersed on carbon with the smallest particle size determined by TEM and XRD.

In the study of Wang et al., the calculated ECSA values of the catalysts were reported as 13.6 m^2^/g and 4.4 m^2^/g for PdCu NCs and Pd black, respectively [14]. Also, Duan et al. calculated the ECSA values for CuPd/C and Pd/C as 22.2 m^2^/g and 14.7 m^2^/g, respectively [19]. Li et al. reported ECSA values for Pt@Pd NCs as 5.62 m^2^/g and Pd black as 3.55 m^2^/g [24]. Therefore, comparable ECSA values were obtained herein, when the reported studies were considered.

**Figure 5:**
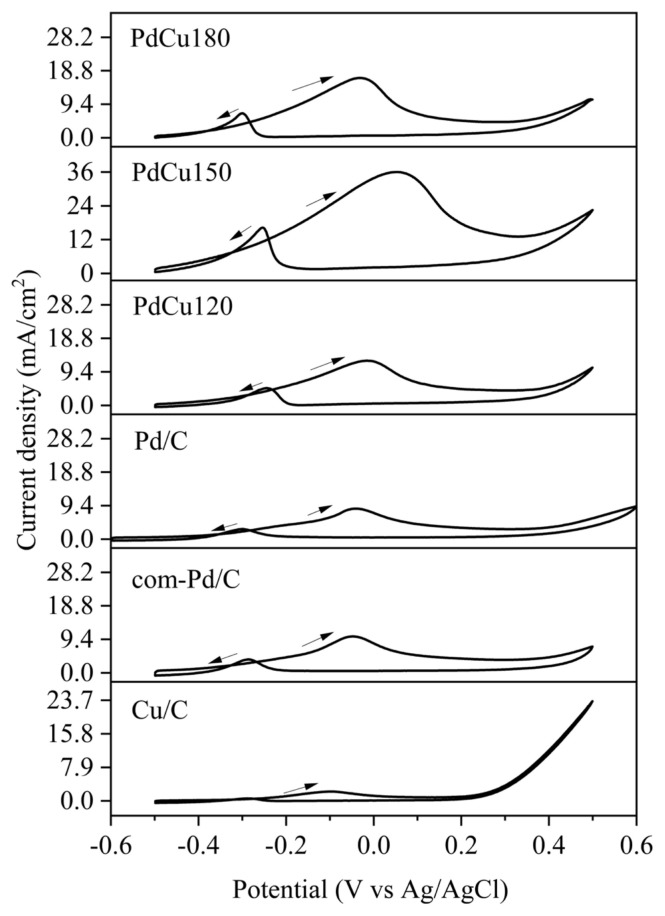
Cyclic voltammogram of electrocatalysts in 0.5 M Glycerol + 0.5 M KOH electrolyte solution with a scan rate of 50 mV/s at 850 rpm.

The electrocatalytic activities of catalysts for glycerol electrooxidation were analyzed between the potentials of −0.6 V and 0.6 V with a scan rate of 50 mV/s in 0.5 M Glycerol +0.5 M KOH solution at 850 rpm. The selection of the medium and the scan rate was due to the information obtained from the literature [4,5,10,24]. The cyclic voltammograms of electrocatalysts are illustrated in Figure 5. As seen, two oxidation peaks were attained in the voltammograms of all electrocatalysts. The observed peak on the forward scan between −0.05 V to 0.05 V and the peak on reverse scan at about −0.27 V were attributed to glycerol oxidation and CO oxidation, respectively [5]. The maximum current densities of glycerol oxidation peak of the catalysts are given in Table 4. As presented, bimetallic PdCu/C electrocatalysts were exhibited much higher current density of oxidation peak compared to other catalysts (Pd/C, Cu/C, and com-Pd/C). As for the GOR activity of PdCu/C, an increase was determined when Cu was added to Pd/C catalyst due to a possible beneficial synergistic effect of PdCu particles. Among bimetallic samples, PdCu150 has the highest current density of 36.02 mA/cm^2^. This may be due to the fact that PdCu150 has the highest electrochemical active area which favors the activity in GOR. Therefore, the microwave duration of 150 s was chosen to suggest high performance and electrochemical surface area. Moreover, the cyclic voltammogram of the Cu/C electrocatalyst in 0.5 M Glycerol +0.5 M KOH exhibited similarity within the 0.5 M KOH solution. This result suggested that there was no significant glycerol electrooxidation activity on Cu/C.

**Figure 6:**
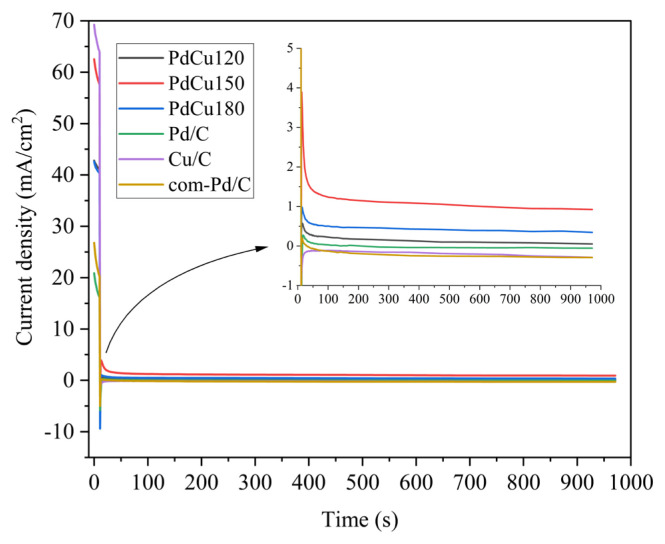
Chronoamperometry curves of electrocatalysts in 0.5 M Glycerol + 0.5 M KOH electrolyte solution for 1000 s.

Chronoamperometric (CA) experiments were conducted to determine the stability of electrocatalysts at −0.4 V for 1000 s in 0.5 M Glycerol +0.5 M KOH electrolyte solution with 850 rpm. All cyclic voltammetry tests were performed between −0.6 V and 0.6 V as shown in Figure 5. Therefore, only the initial stage of oxidation (−0.4 V) was chosen for chronoamperometric experiments. CA curves of all electrocatalysts are depicted in Figure 6. As given, the current densities of electrocatalysts experienced a sharp decrease in the first seconds and stayed stable for 1000 s. The observed decrease at the beginning could be due to rapid poisoning by surface intermediates [10]. When the final current densities of all electrocatalysts were considered, PdCu150 was displayed a higher current density indicating good stability for GOR.

The comparison of glycerol electrooxidation activity with the reported studies in open literature is summarized in Table 5. As seen in Table 5, the current density of PdCu150 is higher than the other electrocatalysts. Furthermore, the modification of traditional polyol method by microwave was favored the electrocatalytic activity for GOR when compared to the study of Maya-Cornejo et al. [9]. Hence, bimetallic PdCu150 electrocatalyst with lower cost could be a suitable candidate for direct glycerol fuel cells.

## 4. Conclusion

In this study, Pd/C, Cu/C, and a series of PdCu/C electrocatalysts were successfully prepared by microwave-assisted modified polyol method and investigated for glycerol electrooxidation reaction (GOR). The physical and electrochemical characterizations of electrocatalysts were conducted and discussed based on activities in GOR. In TEM results, homogeneously dispersed PdCu nanoparticles were determined on the carbon support with a mean particle size of 6.43 nm. The cyclic voltammetry results were shown that glycerol electrooxidation activity was increased with the inclusion of Cu to Pd/C due to a possible synergistic effect between Pd and Cu that is in good agreement with the literature [13,14]. Among as-prepared bimetallic electrocatalysts, PdCu150 was shown the highest activity (36.02 mA/cm^2^) for glycerol electrooxidation compared to PdCu180 (16.77 mA/cm^2^) and PdCu120 (12.54 mA/cm^2^). Therefore, a microwave duration of 150 s during catalyst preparation was found to be favorable on the basis of activity results. Also, the activity of PdCu150 catalyst for GOR was found to be higher than the commercial (com-Pd/C) catalyst (10.23 mA/cm^2^). Recalling that the PdCu150 electrocatalyst was prepared using a novel modified polyol method by microwave, nano-sized, and well-dispersed catalysts with high performance and activity while decreasing the amount of expensive Pd amount, therefore, could be produced. Thus, a conclusion can be drawn that bimetallic PdCu150 electrocatalyst could exhibit a potential for direct glycerol fuel cells taking advantage of high activity, low cost, and good stability.

## Figures and Tables

**Table 1:** Structural properties of electrocatalysts.

Electrocatalyst	Pd 2θ (111)/°	Crystallite size/nm	Pd 2θ (220)/°	Lattice parameter/nm
PdCu120	40.40	3.83	68.53	0.387
PdCu150	40.92	2.85	68.80	0.386
PdCu180	40.97	2.96	69.17	0.384
Pd/C	40.19	6.27	68.10	0.389
com-Pd/C	40.10	9.98	68.09	0.389

**Table 2:** ICP-OES results of electrocatalysts.

Electrocatalyst	Pd loading/wt.%	Cu loading/wt.%	Total metal loadings/wt.%	Pd:Cu atomic ratio
Pd/C	19.7	-	19.7	-
PdCu120	14.5	6.7	21.2	1.3
PdCu150	13.9	7.4	21.3	1.1
PdCu180	13.7	6.6	20.3	1.2

**Table 3:** ECSA results of electrocatalysts.

Electrocatalyst	ECSA (cm^2^/mg)
PdCu120	30.10
PdCu150	140.89
PdCu180	48.13
Pd/C	26.85
com-Pd/C	36.55

**Table 4:** The current densities of electrocatalysts.

Electrocatalyst	Current density (mA/cm^2^)
PdCu120	12.54
PdCu150	36.02
PdCu180	16.77
Pd/C	8.56
Cu/C	2.19
com-Pd/C	10.23

**Table 5:** The comparison of glycerol electrooxidation activities of different electrocatalysts.

Electrocatalyst	Medium	Scan rate (mV/s)	Electrode	Current density	Refs
PtCu/C	0.5 M Glycerol + 0.5 M KOH	10	Carbon cloth	33.5 mA/mg_metal_	[10]
PdCu/C	3 M Glycerol + 0.3 M KOH	50	Glassy carbon	1.39 mA/cm^2^	[9]
Pd black	0.5 M Glycerol + 0.5 M KOH	50	Glassy carbon	9.8 mA/cm^2^	[4]
Pt_1_Au_9_/C	0.1 M Glycerol + 0.1 KOH	20	Glassy carbon	10.9 mA/cm^2^	[22]
Pt@Pd NCs	0.5 M Glycerol + 0.5 M KOH	50	Glassy carbon	28.4 mA/cm^2^	[24]
Pd/C	0.5 M Glycerol + 1 M KOH	1	Glassy carbon	20.9 mA/cm^2^	[25]
PtAg/CNT	0.1 M Glycerol + 1 M KOH	50	Glassy carbon	8.53 mA/μPd	[26]
Pd/CNT	0.1 M Glycerol + 1 M KOH	50	Glassy carbon	2.4 mA/μPd	[26]
PdCu/C	1 M Glycerol + 1 M KOH	30	Gold disk	1.85 mA/cm^2^	[13]
Pd/C	1 M Glycerol + 1 M KOH	30	Gold disk	1.31 mA/cm^2^	[13]
PdCu150	0.5 M Glycerol + 0.5 M KOH	50	Glassy carbon	36.02 mA/cm^2^	This work
Pd/C	0.5 M Glycerol + 0.5 M KOH	50	Glassy carbon	8.56 mA/cm^2^	This work
